# The pentose phosphate pathway of cellulolytic clostridia relies on 6-phosphofructokinase instead of transaldolase

**DOI:** 10.1074/jbc.RA119.011239

**Published:** 2019-12-22

**Authors:** Jeroen G. Koendjbiharie, Shuen Hon, Martin Pabst, Robert Hooftman, David M. Stevenson, Jingxuan Cui, Daniel Amador-Noguez, Lee R. Lynd, Daniel G. Olson, Richard van Kranenburg

**Affiliations:** ‡ Corbion, 4206 AC Gorinchem, The Netherlands; § Thayer School of Engineering, Dartmouth College, Hanover, New Hampshire 03755; ¶ Center for Bioenergy Innovation, Oak Ridge National Laboratories, Oak Ridge, Tennessee 37830; ‖ Cell Systems Engineering, Delft University of Technology, 2629 HZ Delft, The Netherlands; ** Laboratory of Microbiology, Wageningen University & Research, 6708 WE Wageningen, The Netherlands; ‡‡ Department of Bacteriology, University of Wisconsin-Madison, Madison, Wisconsin 53706; §§ Department of Biological Sciences, Dartmouth College, Hanover, New Hampshire, 03755

**Keywords:** pentose phosphate pathway (PPP), phosphofructokinase, mass spectrometry (MS), enzyme kinetics, bacterial metabolism, Clostridium thermocellum, Clostridium thermosuccinogenes, pyrophosphate, sedoheptulose 1,7-bisphosphate, transaldolase

## Abstract

The genomes of most cellulolytic clostridia do not contain genes annotated as transaldolase. Therefore, for assimilating pentose sugars or for generating C_5_ precursors (such as ribose) during growth on other (non-C_5_) substrates, they must possess a pathway that connects pentose metabolism with the rest of metabolism. Here we provide evidence that for this connection cellulolytic clostridia rely on the sedoheptulose 1,7-bisphosphate (SBP) pathway, using pyrophosphate-dependent phosphofructokinase (PP_i_-PFK) instead of transaldolase. In this reversible pathway, PFK converts sedoheptulose 7-phosphate (S7P) to SBP, after which fructose-bisphosphate aldolase cleaves SBP into dihydroxyacetone phosphate and erythrose 4-phosphate. We show that PP_i_-PFKs of *Clostridium thermosuccinogenes* and C*lostridium thermocellum* indeed can convert S7P to SBP, and have similar affinities for S7P and the canonical substrate fructose 6-phosphate (F6P). By contrast, (ATP-dependent) PfkA of *Escherichia coli*, which does rely on transaldolase, had a very poor affinity for S7P. This indicates that the PP_i_-PFK of cellulolytic clostridia has evolved the use of S7P. We further show that *C. thermosuccinogenes* contains a significant SBP pool, an unusual metabolite that is elevated during growth on xylose, demonstrating its relevance for pentose assimilation. Last, we demonstrate that a second PFK of *C. thermosuccinogenes* that operates with ATP and GTP exhibits unusual kinetics toward F6P, as it appears to have an extremely high degree of cooperative binding, resulting in a virtual on/off switch for substrate concentrations near its *K*_½_ value. In summary, our results confirm the existence of an SBP pathway for pentose assimilation in cellulolytic clostridia.

## Introduction

**Figure 1.**
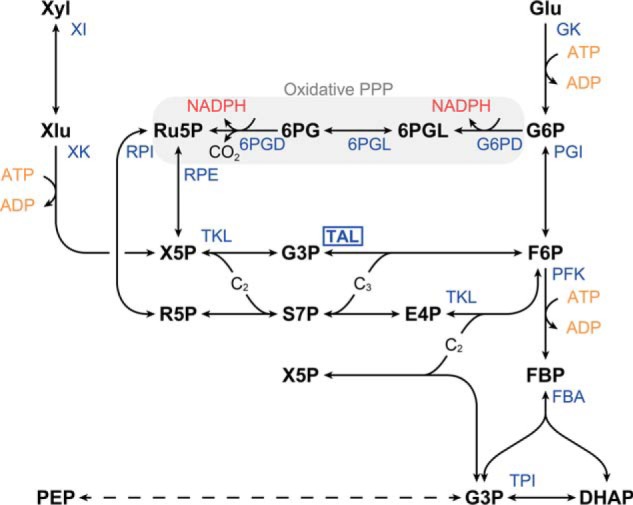
**The reactions of the pentose phosphate pathway and its connection to the C_3_/C_6_ metabolites of the Embden-Meyerhof-Parnas (*EMP*) pathway.** *6PG*, 6-phosphogluconate; *6PGD*, 6-phosphogluconate dehydrogenase; *6PGL*, 6-phosphogluconolactone; *FBA*, fructose bisphosphate aldolase; *GK,* glucokinase; *Glu*, glucose; *G6P*, glucose 6-phosphate; *G6PD*, glucose-6-phosphate dehydrogenase; *P_i_*, orthophosphate; *PEP*, phosphoenolpyruvate; *PGI*, phosphoglucoisomerase; *PP_i_*, pyrophosphate; *R5P*, ribose 5-phosphate; *RPI*, ribose 5-phosphate isomerase; *Ru5P*, ribulose 5-phosphate; *RPE*, ribulose 5-phosphate 3-epimerase; *TAL*, transaldolase; *TKL*, transketolase; *TPI*, triose-phosphate isomerase; *Xyl,* xylose; *XI*, xylose isomerase; *XK*, xylulokinase; *Xlu*, xylulose; *X5P*, xylulose 5-phosphate. The *dashed arrow* represents the trunk or lower part of the glycolysis, which consist of several reactions. *X5P* is shown in two different locations in the diagram for clarity. The *gray box* indicates reactions in the oxidative PPP.

Transaldolase plays a key role in the non-oxidative pentose phosphate pathway (PPP).^3^ Together with transketolase it is responsible for the interconversion of C_5_ and C_3_/C_6_ metabolites, as depicted in Fig. 1. Specifically, transaldolase transfers a three-carbon ketol unit from sedoheptulose 7-phosphate (S7P) to glyceraldehyde 3-phosphate (G3P), forming erythrose 4-phosphate (E4P) and fructose 6-phosphate (F6P). Transketolase is responsible for the transfer of a two-carbon ketol unit from xylulose 5-phosphate, either to ribose 5-phosphate, yielding the S7P and G3P used by transaldolase, or to E4P, one of the products of transaldolase, yielding G3P and F6P. In contrast to the oxidative part, the non-oxidative PPP is reversible and essential both for catabolism of pentoses (*e.g.* xylose) and for the production of the C_5_ metabolites required for anabolism during growth on other substrates. The latter can also be facilitated by the oxidative PPP, but is then accompanied with the formation of NADPH, another important role of the PPP in many organisms.

**Figure 2.**
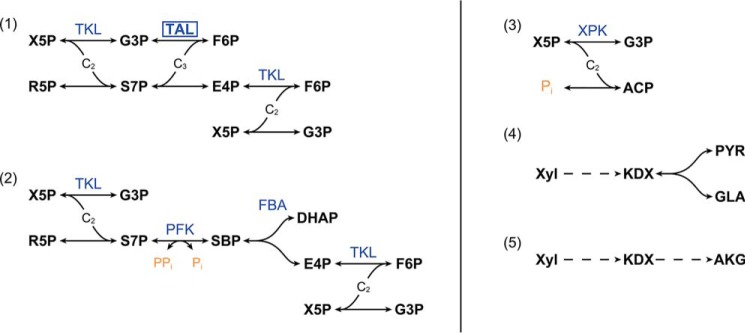
**Overview of the different pathways known for the interconversion of C_5_ and C_3_/C_6_ metabolites.** (*1*) Typical pentose phosphate pathway involving transketolase and transaldolase. (*2*) Sedoheptulose 1,7-bisphosphate pathway. (*3*) Phosphoketolase pathway. (*4*), Dahms pathway. (*5*) Weimberg pathway. *ACP*, acetyl phosphate; *AKG*, α-ketoglutarate; *FBA*, fructose-bisphosphate aldolase; *GLA*, glycolaldehyde; *KDX*, 2-keto-3-deoxy-d-xylonate; *PP_i_*, pyrophosphate; *PYR*, pyruvate; *R5P*, ribose 5-phosphate; *TAL*, transaldolase; *TKL*, transketolase; *X5P*, xylulose 5-phosphate; *XPK*, xylulose 5-phosphate phosphoketolase; *Xyl*, xylose. *Dashed arrows* represent more than one reaction.

Several cellulolytic clostridia^4^ have been reported to lack an annotated transaldolase gene, whereas at least a few of those are able to grow very efficiently on pentose sugars, including *Clostridium thermosuccinogenes* and *Clostridium cellobioparum* subsp. *termitidis*, and *Clostridium stercorarium* (1–3). This implies that an alternative route to link C_5_ to the rest glycolysis must be present in those organisms. In *C. thermosuccinogenes* only the genes in the PPP responsible for the conversions of C_5_ sugars to xylulose 5-phosphate (*i.e.* xylose transporters, xylose isomerase, and xylulokinase) were up-regulated during growth on xylose *versus* glucose (1). In *Clostridium termitidis*, a transketolase was found to be up-regulated as well, during growth on xylose or xylan *versus* cellobiose (2). Yet, neither of those transcriptome studies resulted in an obvious candidate for an alternative pathway. *Clostridium thermocellum* also lacks an annotated transaldolase, but in contrast to the other cellulolytic clostridia, it cannot grow on xylose (4). However, it lacks the oxidative PPP as well (present in *C. thermosuccinogenes*) (5), so *C. thermocellum* would still be expected to have an alternative route, to produce the C_5_ metabolites required for anabolism. Although most organisms use transketolase/transaldolase, several alternative pathways to convert C_5_ to C_3_/C_6_ intermediates are known to exist (Fig. 2).

- The phosphoketolase pathway (PKP). In the PKP, xylulose 5-phosphate is directly cleaved with orthophosphate into acetyl-P and G3P by phosphoketolase (6). The PKP is responsible for the degradation of pentose sugars in lactic acid bacteria, but more recently, it was shown that PKP is also, at least in part, responsible for pentose utilization in a variety of other bacteria, including *Clostridium acetobutylicum* and cyanobacteria (7–9).
- The Weimberg pathway and Dahms pathway. The Weimberg pathway is a 5-step, oxidative, nonphosphorylating pathway that converts pentoses into 2-oxoglutarate, an intermediate in the TCA cycle (10, 11). In the Dahms pathway 2-keto-3-deoxy-d-xylonate or 2-keto-3-deoxy-l-arabinonate, intermediates in the Weimberg pathway, are cleaved by an aldolase into pyruvate and glycolaldehyde (11, 12). Both variants of the pathway occur in prokaryotes.
- The sedoheptulose 1,7-bisphosphate pathway. In the amoebozoan *Entamoeba histolytica*, which lacks both glucose 6-phosphate dehydrogenase and transaldolase, S7P was shown to be the substrate of a pyrophosphate (PP_i_)-dependent 6-phosphofructokinase (PFK). The resulting sedoheptulose 1,7-bisphosphate (SBP) is subsequently cleaved into dihydroxyacetone phosphate (DHAP) and E4P by fructose-bisphosphate aldolase, effectively replacing the function of transaldolase, shown in Fig. 2 (13). PP_i_-PFK is physiologically reversible, in contrast to ATP-dependent PFK, allowing it to function in the PPP. Although, a number of reports exists where S7P kinase activity with high affinity is documented for PPi-PFKs from methanotrophs (14–17), the study discussed above is the only one where the SBP pathway was shown to exist in a WT metabolism. A double transaldolase knockout in *Escherichia coli* (Δ*talAB*) resulted in xylose degradation via the SBP pathway, in conjunction with its native ATP-PFK and fructose-bisphosphate aldolase enzymes. In addition, S7P and SBP were observed to accumulate (18).

There is no real indication (genomic or physiological) for any of the routes other than the SBP pathway, based on the reliance of cellulolytic clostridia on PP_i_-PFKs, analogous to *E. histolytica*. Hence, the SBP pathway is generally assumed to be the responsible pathway (19, 20), but this has never been experimentally verified. This is not a trivial exercise, as metabolites required for enzyme assays are difficult to acquire and stable isotopic labeling studies are complicated by the recursive nature of the PPP and the typically low thermodynamic driving force in anaerobic metabolism, leading to relative high reverse fluxes (21, 22).

SBP should be a rather uncommon metabolite in bacteria without the Calvin cycle, the only known pathway with SBP as intermediate. In this cycle, SBP is formed from E4P and DHAP by aldolase and cleaved by sedoheptulose bisphosphatase. Besides algae and plants, some nonphotosynthetic eukaryotes have also been reported to possess this enzyme (23), but it has not been annotated in any of the cellulolytic clostridia. Therefore, presence of an SBP pool alone in *C. thermosuccinogenes* would already be a strong, albeit indirect, indication for the presence of the SBP pathway. The first aim of this study was therefore to investigate the possible presence of SBP in *C. thermosuccinogenes*, using high mass resolution Orbitrap MS. Unfortunately, an SBP reference standard was not commercially available, due to poor chemical stability. Instead, we constructed an *E. coli* Δ*talAB* strain with a double transaldolase knockout, which accumulated SBP (18). We were able to use metabolite extracts from this strain as an SBP reference.

The accumulation of S7P in addition to SBP in *E. coli* Δ*talAB* suggests that *E. coli* PFK has a low affinity for S7P. However, this is expected, because activity toward SBP is not necessary in *E. coli* because of the presence of transaldolase. If the PP_i_-PFKs of cellulolytic clostridia are indeed natively responsible for the conversion of S7P in the PPP, one would expect a much higher affinity for S7P. Therefore, the second aim of the study was to confirm *in vitro* the ability of PFKs of phosphorylate S7P and to compare their affinity for S7P *versus* F6P.

## Results

### Metabolomics

Presence of SBP in metabolite extracts of cellulolytic clostridia would give a preliminary indication of the presence of the SBP pathway. To unambiguously confirm the presence of SBP it is crucial to have a reference standard. Nakahigashi *et al.* (18) showed the accumulation of S7P and a molecular ion conform with SBP (*m*/*z* 369.0) in extracts of *E. coli* harboring a double transaldolase knockout (Δ*talAB*). We decided to use the above mentioned strain, which presumably accumulated SBP as well as S7P, to subsequently function as a molecular reference for SBP. The strain used by Nakahigashi *et al.* (18) was recreated as described under “Materials and methods.” The growth rate of the Δ*talAB* derivative on a minimal medium with xylose was only marginally lower compared with that of the WT: 0.33 ± 0.01 h^−1^ *versus* 0.39 ± 0.01 h^−1^, when grown in shake flasks with 50 ml of M9 medium (in triplicate, with the standard deviation reported), in line with the findings of Nakahigashi *et al.* (18). Furthermore, the growth rate on minimal medium with glucose was in fact slightly higher for the mutant: 0.52 ± 0.01 h^−1^ *versus* 0.49 ± 0.01 h^−1^.

**Figure 3.**
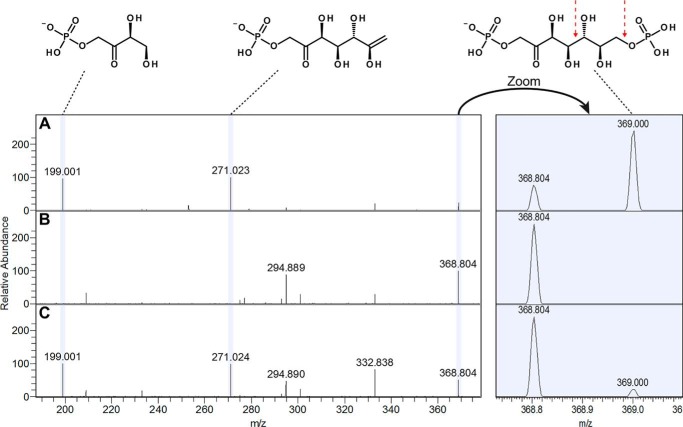
**Identification of SBP (*m*/*z* 368.99) via targeted monitoring of fragments for the precursor ion at *m*/*z* 368–370.** *A, E. coli* Δ*talAB* extract; *B,* blank run; *C,* xylose grown *C. thermosuccinogenes* extract. A clear peak is present in *A* and *C* at *m*/*z* 369 corresponding to SBP, confirmed by indicative fragment peaks at *m*/*z* 199 and 271, which are further absent in the blank (*B*). The *red arrow* indicates the bonds that, when broken, result in the fragment at *m*/*z* 199 and 271. The identity of the peak at *m*/*z* 368.804 is unknown. Presence of this peak in the blank run (*B*) indicates that it is a background signal, whereas the nearby peak at *m*/*z* 369 is not.

Mass spectrometry of metabolome extracts from *E. coli* Δ*talAB* and WT grown on xylose showed the accumulation of S7P and a compound with a *m*/*z* 369.0 for the Δ*talAB* strain. This peak showed (i) the expected accurate mass of SBP, (ii) the retention behavior relative to S7P corresponding to the chemical composition of SBP (*i.e.* slightly later and with a higher tendency for tailing, due to higher acidity), and (iii) the higher-energy collisional dissociation fragments exactly matching those expected for SBP (*i.e.* phosphate ester loss and β-bond cleavage), as shown in Fig. 3. Based on these observations it was concluded that SBP was indeed produced and that it could successfully be used as a reference.

Nevertheless, we could only successfully detect SBP at a relatively strong signal (comparable to S7P), when the metabolic extract was analysed without any preceding purification/enrichment steps, further illustrating its very low stability.

Traces of SBP were also detected in the cell extract of WT *E. coli*, suggesting that even in the WT metabolism a small fraction of the S7P is converted to SBP by PFK. This flux is amplified after the double transaldolase deletion, causing S7P to accumulate. This provides further support for our assumption that the *E. coli* PFKs have a low affinity for S7P.

**Figure 4.**
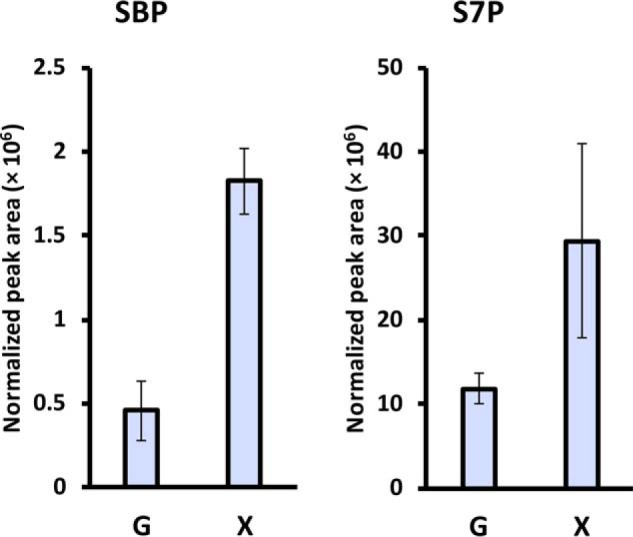
**Relative pools of SBP and S7P in *C. thermosuccinogenes* grown on xylose (*X*), compared with glucose (*G*).** Mass spectrometry peak area is normalized to OD_600_ of the culture. The *error bars* represent the mean ± S.D. of biological triplicates.

Next, *C. thermosuccinogenes* was grown on xylose *versus* glucose, to try to detect SBP, and determine if the SBP pool increases during growth on xylose, as would be expected, because virtually the entire flux of carbon will have to be channeled through SBP into glycolysis. The results of the metabolome extract analysis are shown in Fig. 4. SBP was found to be present and normalized to the optical density of the cultures at 600 nm (OD_600_), the SBP concentration increased 4-fold. Similarly, the S7P concentration increased 2.5-fold during growth on xylose.

In *E. coli* Δ*talAB* grown on xylose, the S7P concentration was roughly 6-fold higher compared with that of *C. thermosuccinogenes* grown on xylose. For SBP this difference was roughly 20-fold. Although many factors could explain the difference in concentrations between the two organisms, the higher accumulation in *E. coli* suggests that the PFK and the fructose-bisphosphate aldolase enzymes of *C. thermosuccinogenes* have higher affinities for S7P and SBP, respectively, compared with those of *E. coli*. A higher affinity, in turn, suggests evolutionary pressure toward the use of those substrates. For this reason, we studied the *in vitro* affinities of the cellulolytic clostridia PFKs toward F6P and S7P.

### In vitro phosphofructokinase assays

*C. thermosuccinogenes* and *C. thermocellum* contain two PFKs: the PP_i_-dependent PFK and another one that was shown to function with both ATP and GTP in *C. thermosuccinogenes* (24). A third PFK has been annotated in the genome of *C. thermosuccinogenes* for which no activity had been detected, and which is absent in *C. thermocellum*. From assays with cell-free extract, it was already clear that PP_i_-dependent PFK is the dominant isoform (1, 25). We repeated the aforementioned assays with *C. thermosuccinogenes* cell-free extract in the presence of NH_4_, which we had serendipitously found to activate ATP/GTP-PFK, but were still unable to detect ATP-dependent activity (data not shown), confirming that PP_i_-dependent PFK is the dominant isoform in this organism as well.

**Figure 5.**
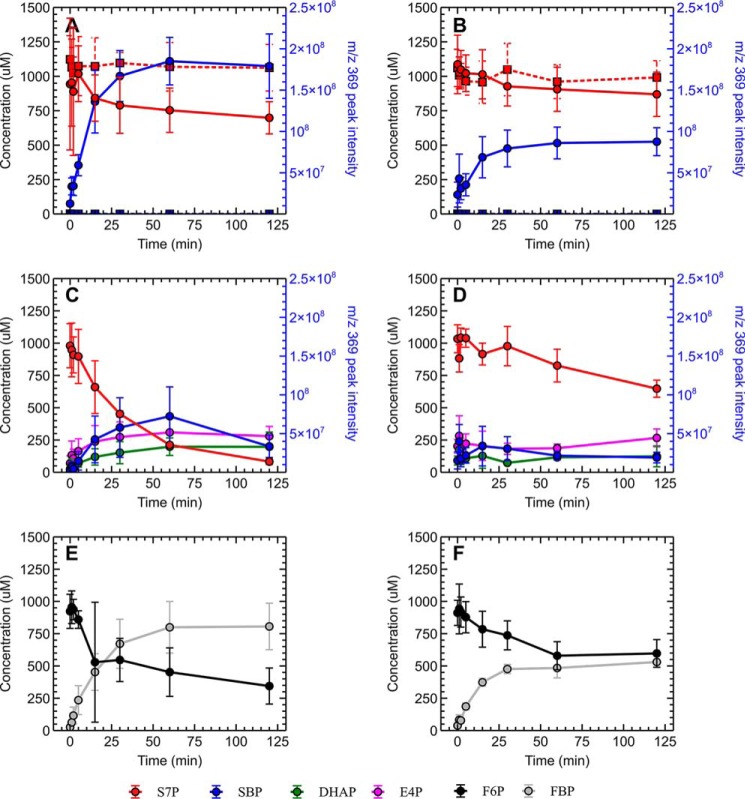
***In vitro* time course assay of *C. thermocellum* (*A*, *C*, and *E*) and *C. thermosuccinogenes* (*B*, *D*, and *F*) PP_i_-PFK proteins' abilities to convert S7P to SBP.** *A* and *B,* conversion of S7P (*red*) in the presence (*circled data points* on *solid lines*) or absence (*square data points* on *dotted lines*) of 5 mm pyrophosphate, with corresponding increase in a compound (SBP) with a *m*/*z* of 369 (*blue*). SBP peak intensities in assays lacking PP_i_ were, in general, between the range of 10,000 and 20,000 arbitrary units throughout the assay. *C* and *D,* conversion of S7P (*red*) to SBP (*blue*), and SBP's subsequent conversion to DHAP (*green*) and E4P (pink). *E* and *F,* control reactions for the purified PP_i_-PFK proteins, demonstrating their ability to function as 6-PFKs and convert F6P (*black*) to FBP (*gray*). *Error bars* represent 1 S.D. (*n* ≥ 2).

To determine whether the PP_i_-PFK proteins participated in the non-oxidative pentose phosphate pathways of their respective organisms, we investigated whether the PP_i_-PFKs were in fact capable of interconversion of S7P and SBP. *In vitro* time course experiments showed that S7P concentrations decreased over time in an assay mixture where both PP_i_-PFK and PP_i_ were also present (Fig. 5); this decrease in S7P concentrations was concomitant with an increase in signal intensity at the *m*/*z* 369, which corresponds to the presence of SBP (Fig. 3). In the absence of PP_i_, S7P concentrations remained relatively stable, and no increase in signal intensity at *m*/*z* 369 was observed (Fig. 5), providing further evidence that the PP_i_-PFK proteins were using PP_i_ as a cofactor to phosphorylate of S7P. As expected, assay reactions containing S7P and PP_i_, but no PP_i_-PFK protein, did not show any decrease in S7P, nor increase in peak intensity at *m*/*z* 369 (Fig. S1).

Further confirmation of the identity of the *m*/*z* 369 compound as SBP was obtained by repeating the assay with the inclusion of fructose-bisphosphate aldolase. The SBP pathway would result in the formation of E4P and DHAP (both commercially available compounds) from SBP via the action of the aldolase (Fig. 2). In this additional set of assays, a similar pattern of decreasing the S7P concentration coupled to an increase in peak intensity at *m*/*z* 369 was observed; in addition, it was also observed that E4P and DHAP concentrations increased over time as well, where accumulation of these compounds was not detected in the reactions without the added aldolase. Notably, the peak intensities at *m*/*z* 369 in the aldolase-containing reactions were lower than that observed in the corresponding assay reactions that did not contain aldolase, in line with the conversion of SBP to E4P and DHAP. Furthermore, S7P concentrations in the aldolase-containing reactions trended to be lower than in their corresponding aldolase-free reactions; this is likely due to the consumption of SBP by aldolase, producing DHAP and E4P, which promotes further conversion of S7P to SBP by delaying the chemical equilibrium. It was also observed that the DHAP concentrations tended to be lower than those of E4P, despite the fact that they should be produced in equimolar amounts, as the stoichiometry in Fig. 2 would suggest; one explanation is that the added rabbit aldolase contains triose-phosphate isomerase as a trace contaminant, which would catalyze the interconversion of DHAP to G3P. Nonetheless, the results support the proposed SBP pathway.

**Figure 6.**
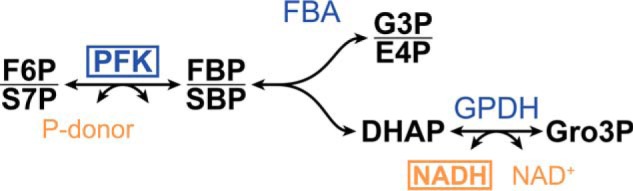
**Enzyme assay to couple FBP/SBP formation by PFK to NADH oxidation, allowing the study of the PFK enzyme kinetics.** *FBA*, fructose-bisphosphate aldolase; *GPDH*, glycerol-3-phosphate dehydrogenase; *FBP*, fructose 1,6-bisphosphate; *G3P*, glyceraldehyde 3-phosphate; *Gro3P*, glycerol 3-phosphate. *Boxes* highlight the investigated enzyme (*i.e.* PFK) and detected metabolite (*i.e.* NADH).

For the determination of the enzyme kinetics for F6P and S7P, analysis via MS is impractical, as the response is not obtained in real-time. Instead, the formation of FBP and SBP can be coupled to oxidation of NADH via auxiliary fructose-bisphosphate aldolase and glycerol-3-phosphate dehydrogenase (both from rabbit), as illustrated in Fig. 5. We confirmed that rabbit aldolase was able to convert the formed SBP to E4P and DHAP, validating the coupled assay method, as shown in Fig. 6. Note that commonly, triose-phosphate isomerase is used additionally for such assays (to convert glyceraldehyde 3-phosphate to DHAP, increasing the signal and the driving force), which we excluded, as this would not function with E4P, making it easier to directly compare the two different substrates.

The results of the kinetics assays are presented in Table 1. For clarity, we use the term *affinity* to discuss *K_m_* values, although strictly speaking this is incorrect, as *K_m_* is not equal to the dissociation constant. Although the two tested PP_i_-dependent PFKs showed 2–3-fold lower maximal activity (*k*_cat_) with S7P *versus* F6P, the affinity (*K_m_*) for both substrates was comparably high (*K_m_* of ∼0.1 mm). This is in stark contrast with the PfkA from *E. coli*, for which the affinity constant for S7P is almost 2 orders of magnitude larger (*i.e.* lower affinity; *K_m_* of 2.5 mm) than that for F6P; the latter being on par with the affinities of the other tested PFKs. In exponentially growing *E. coli* cells on glucose, the concentrations of F6P and S7P are 2.2 and 0.9 mm, respectively (26), which means that *in vivo* PfkA is highly saturated with F6P, but not with S7P. The intracellular concentrations of those metabolites are not known in any of the cellulolytic clostridia, but with the equally high affinities for both F6P and S7P it is reasonable to assume that both metabolites are saturating, and thus physiologically relevant substrates for the PP_i_-dependent PFKs. These results strongly suggest that the PP_i_-dependent PFKs of cellulolytic clostridia, lacking a transaldolase, evolved for the use of S7P as a substrate, whereas the (ATP-dependent) PfkA of *E. coli*, which does possess a transaldolase, did not; although PfkA is still able to use S7P as a substrate at higher, nonphysiological concentrations. The latter can explain why traces of SBP were found in WT *E. coli* grown on xylose, and why S7P accumulates in the Δ*talAB* strain. The data of the assays and the fitted kinetic models can be found in Figs. S2–S5.

**Table 1:** **Kinetics of the PFKs tested** Parameters of Michaelis-Menten (or Hill) kinetics were approximated by minimizing the sum of the squared vertical difference. The plots with the data points can be found in Figs. S2–S5. ATP/GTP-PFK did not show any activity with S7P, and due to the high cooperativity (*n* = 24) with F6P, it was not possible to fit the Michaelis-Menten equation.

			*K_m_*	*k*_cat_	*n*	*k*_cat_/*K_m_*
			*mm*	*s*^−*1*^		*s*^−*1*^ *m*^−*1*^
CDQ83_11320 **C.ts PP*_i_*-PFK**	PP_i_	F6P	0.070	182	NA*^a^*	2.6 × 10^6^
		S7P	0.11	49		0.46 × 10^6^
Clo1313_1876 **C.tc PP*_i_*-PFK**		F6P	0.046	127	NA	2.8 × 10^6^
		S7P	0.10	80		0.80 × 10^6^
CDQ83_07225 **C.ts A/GTP-PFK**	ATP	F6P	0.688	41	24	59 × 10^3^
		S7P	–	–	–	–
PfkA **E.co PFK**		F6P	0.065	1.2	NA	19 × 10^3^
		S7P	2.5	0.25		0.15 × 10^3^

*^a^* NA, not applicable. The Hill equation is only used in the case of CDQ83_07225, where *n* is the Hill coefficient, and *K_m_* is replaced by *K*_½_.

**Figure 7.**
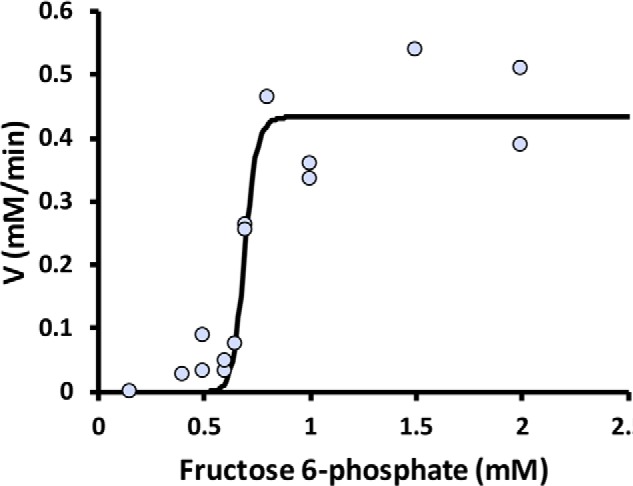
**Fitted Hill kinetics to the results of the coupled assays with ATP/GTP-PFK (CDQ83_07225) of *C. thermosuccinogenes*.** *V* represents the rate of NADH oxidation per minute, which is plotted against the corresponding fructose 6-phosphate concentration.

The ATP/GTP-dependent PFK of *C. thermosuccinogenes* was able to use F6P, but did not show any activity with S7P, at least, not in the range of tested S7P concentrations, up to 4 mm. Interestingly, it showed an extreme degree of cooperativity with F6P, reflected by a Hill coefficient (*n*) of 24. The high degree of cooperativity results in a virtual on/off switch of the enzyme, activating it at F6P concentrations above the *K*_½_ of 0.7 mm, as can be seen in Fig. 7.

## Discussion

### The SBP pathway in cellulolytic clostridia

A considerable pool of SBP is shown to be present in *C. thermosuccinogenes*, which, together with the S7P pool, increases severalfold when *C. thermosuccinogenes* is grown on xylose *versus* glucose. This increase demonstrates the role for SBP in the pentose metabolism, and agrees with the hypothesis that the SBP pathway is present instead of transaldolase. In the SBP pathway, PFK and fructose-bisphosphate aldolase together convert S7P to E4P and DHAP (via SBP, as shown in Fig. 2). These two enzymes are known to convert F6P to glyceraldehyde 3-phosphate and DHAP (via fructose 1,6-bisphosphate as intermediate). *In E. coli*, it was already shown that these enzymes could take over the role of transaldolase after a double transaldolase knockout, and in *E. histolytica* it was shown that the SBP pathway likely exists in the WT metabolism (13, 18).

If in *C. thermosuccinogenes* and other cellulolytic clostridia, in the absence of a transaldolase, the SBP pathway is really the native pathway to connect pentose with hexose metabolism, their affinities for these “alternative” substrates should reflect that. Indeed, here we show that the PP_i_-PFKs of both *C. thermosuccinogenes* and *C. thermocellum* can use S7P, and have an affinity similar to that for F6P. The same was previously found for *E. histolytica* PP_i_-PFK, where the *K_m_* for S7P is 0.064 mm and 0.038 mm for F6P (13). On the contrary, here we show that the affinity of *E. coli* PfkA for S7P is almost 2 orders of magnitude lower compared with its affinity for F6P, such that the affinity constant for S7P is much higher than its typical intracellular concentration. Considering the fact that *E. coli* has a transaldolase to facilitate the interconversion of pentoses and hexoses, it makes sense that the affinity of PfkA for S7P is such that *in vivo* this reaction does not proceed; there is no need for S7P kinase activity and the resultant SBP pathway.

### The SBP pathway versus transaldolase

A question that remains is whether there is an advantage to having either the transaldolase or the SBP pathway? A crucial aspect of the SBP pathway is the physiological reversibility of the PP_i_-PFK in contrast to the ATP-dependent variant (27), because the non-oxidative PPP should be able to operate in both directions. It therefore seems a prerequisite to rely on PP_i_-PFK, and the associated PP_i_-generating metabolism to use the SBP pathway. If this is not the case and PFK is ATP-dependent, transaldolase would still be required to facilitate the reverse (anabolic) direction in the PPP. As such, it seems that transaldolase might simply be or become obsolete in the case PP_i_-PFK is used in glycolysis. The underlying question therefore is why a PP_i_-dependent PFK is used at all, instead of an ATP-dependent one?

The irreversibility (*i.e.* large decrease in Gibbs free energy) of ATP-dependent PFK grants it a lot of control over the metabolism, but comes at the cost of about half-available free energy (26). The trade-off between control and energy conservation could perhaps be the main factor behind the use of a PP_i_-dependent PFK *versus* an ATP-dependent one. Organisms that almost exclusively rely on substrate level phosphorylation for ATP generation, such as the strictly anaerobic cellulolytic clostridia, might prioritize energy conservation over control, whereas respiring organisms might have done the opposite.

PP_i_ is a by-product of many anabolic reactions, often operating close to equilibrium. Many organisms hydrolyze PP_i_ to orthophosphate using inorganic pyrophosphatase, to drive these anabolic reactions forward, releasing heat (28). Using the otherwise “wasted” PP_i_ instead of ATP for the phosphorylation of F6P should therefore allow the conservation of metabolic energy. It was already calculated for *C. thermocellum*, however, that the formation of PP_i_ as by-product of anabolism alone is not enough to sustain the PFK reaction in glycolysis as it accounted for less than 10% of the flux (25), meaning that there must be another, dedicated source of PP_i_.

### Unusual kinetics for ATP/GTP-PFK

ATP/GTP-PFK was previously found to have similar affinities for ATP *versus* GTP (24), and here we show that it has an extreme degree of cooperativity for F6P. The extreme cooperativity effectively means that below ∼0.7 mm F6P there is no activity, whereas above this concentration the enzyme operates at maximum activity. At this point we can only speculate on the function behind this peculiar characteristic, and doing so it seems wholly plausible that it could function as a kind of relief valve that prevents the intracellular concentration of F6P from rising above 0.7 mm.

It might in fact be detrimental for organisms relying on the SBP pathway to accumulate a large intracellular concentration of F6P (relative to S7P/SBP), due to the competition between S7P/SBP and F6P/fructose 1,6-bisphosphate for PP_i_-PFK. For example, a 10-fold higher F6P concentration compared with S7P means that only ∼9% of the PP_i_-PFK enzyme pool is available for S7P-depdendent activity. The other way around, when S7P accumulates relative to F6P, which prevents F6P phosphorylation by PP_i_-PFK, presence of ATP/GTP-PFK will still enable this reaction to occur, but with ATP or GTP instead of PP_i_.

In the case one enzyme is responsible for two separate (metabolic) reactions (via the same active site), it becomes crucial for the cell to regulate those relative pools, for both reactions to be able to occur. Our hypothesis is that the ATP/GTP-dependent PFK is responsible for a fail-safe mechanism relieving the negative effects caused by the perturbation of the S7P and F6P pools. How and if the enzyme's activation by NH_4_^+^ relates to this hypothesized function is not clear.

### Widespread occurrence of the SBP pathway?

It is common for both PP_i_-dependent and ATP-dependent PFKs to coexist in one organism. In such cases, it was previously thought that PP_i_-PFK might have an alternative, unknown function (29). Here we show that the PP_i_-PFK has a dual function in glycolysis and the PPP, whereas the ATP-dependent PFK might have an alternative function. Similarly, in *Amycolatopsis methanolica* PP_i_-PFK is used in glycolysis during growth on glucose, whereas its ATP-PFK is used in the ribulose monophosphate cycle, during growth on one-carbon compounds (30). The widespread occurrence of PP_i_-PFK could therefore suggest that the SBP pathway is also more widespread than is currently recognized, particularly when the presence of PP_i_-PFK coincides with the absence of a transaldolase. The latter might also be underestimated due to F6P aldolases being wrongly annotated as transaldolase, resulting from their high similarity (31, 32).

Of all the 45 cellulolytic clostridia (*Hungateiclostridiaceae*) genomes in the JGI database, only the two *Ruminiclostridium papyrosolvens* genomes contain annotated transaldolases; two per genome, of which one contains the characteristic Glu and Tyr residues associated with transaldolase activity, rather than F6P aldolase activity (32). Except for the *C. thermosuccinogenes* genomes, none of the genomes harbors a complete oxidative PPP, and besides the *C. thermocellum* genomes almost all harbor xylose isomerase and xylulokinase (required for growth on pentoses), meaning that cellulolytic clostridia in general rely on the SBP pathway for the PPP, with the possible exception of *R. papyrosolvens*.

How widespread the SBP pathway is outside the cellulolytic clostridia would require further research, which is outside the scope of this study. The spread of PP_i_-PFKs in a wide variety of organisms, the proved existence of the SBP pathway in cellulolytic clostridia as well as the eukaryotic *E. histolytica*, and the high affinity of methylotrophic PFKs for S7P does hint at a much more widespread occurrence of the pathway.

### SBP identification

In some metabolomics studies, the identification of SBP is simply omitted, because the standard reagent was not available (33). In others, SBP was synthesized *in vitro* using purified enzymes (34), which is laborious and expensive; and due to the low stability the product cannot be stored for longer periods. The method described here for the identification of SBP, relying on the *E. coli* Δ*talAB* strain is simple and cheap, and might therefore benefit other researchers studying pentose metabolism. Furthermore, we noticed that commonly used practices for metabolomics studies, such as prolonged storage, and enrichment of extracts (*e.g.* via vacuum evaporation) will decrease the chance of detecting SBP in the extracts enormously.

## Conclusion

An *E. coli* double transaldolase mutant was shown here to accumulate SBP, verified by Orbitrap MS. A metabolite extract from this *E. coli* mutant was used as an SBP reference for analysis (because SBP is not commercially available), and enabled us to show that a significant pool of SBP is present in *C. thermosuccinogenes*, an uncommon metabolite in organisms without the Calvin cycle. Moreover, the SBP pool was elevated during growth on xylose, confirming its relevance in pentose assimilation.

*In vitro* assays showed that PP_i_-PFK of *C. thermosuccinogenes* and *C. thermocellum* is able to convert S7P to SBP, and that they have similar affinity for S7P and F6P, the canonical substrate. In contrast, PfkA of *E. coli* showed a very poor affinity for S7P, which explains the high accumulation of S7P and SBP in the *E. coli* mutant. Furthermore, the enzyme kinetics suggest that the PP_i_-PFK enzymes of cellulolytic clostridia may have evolved for the use of S7P.

Additionally, we found that the ATP/GTP-dependent PFK of *C. thermosuccinogenes* shows an extremely high degree of cooperative binding toward F6P, resulting in a virtual on/off switch for substrate concentrations near its *K*_½_ value. We hypothesize that this PFK might represent a fail-safe mechanism that regulates the relative pools of F6P and S7P to prevent competition for the active site of PP_i_-PFK between the two parallel substrate pools causing one substrate to dominate the other. Overall, these results verify the existence of the SBP pathway in cellulolytic clostridia instead of the canonical transaldolase, connecting pentose metabolism with the rest of the metabolism.

## Materials and methods

### Growth medium and cultivation

For strain construction, plasmid construction, and protein purification, *E. coli* strains were grown on LB medium containing per liter 10 g of tryptone, 5 g of yeast extract, 10 g of NaCl.

For metabolome extraction, *E. coli* BW25113 was grown on M9 minimal medium, made with M9 Minimal Salts (×5) from Sigma, containing additionally 0.4% xylose, 1 mm MgSO_4_, 0.3 mm CaCl_2_, 1 mg/liter of biotin, and 1 mg/liter of thiamine hydrochloride, which were all separately sterilized. Cells were grown at 37 °C in shaker flasks containing 50 ml of medium, inoculated with 0.5 ml of overnight culture grown in LB.

*C. thermosuccinogenes* was grown anaerobically in adapted CP medium (35), as described previously (1), which contained per liter 0.408 g of KH_2_PO_4_, 0.534 g of Na_2_HPO_4_·2H_2_O, 0.3 g of NH_4_Cl, 0.3 g of NaCl, 0.1 g of MgCl_2_·6H_2_O, 0.11 g of CaCl_2_·2H_2_O, 4.0 g of NaHCO_3_, 0.1 g of Na_2_SO_4_, 1.0 g of l-cysteine, 1.0 g of yeast extract (BD Pharmingen, BD Bacto), 1 ml of vitamin solution, 1 ml of trace elements solution I, and 1 ml of trace elements solution II. No resazurin was added to eliminate the possibility of it interfering with the metabolomics, as it appeared to adsorb to the nylon filter used for making the metabolome extracts.

The vitamin solution, which was ×1,000 concentrated, contained per liter 20 mg of biotin, 20 mg of folic acid, 100 mg of pyridoxine-HCl, 50 mg of thiamine-HCl, 50 mg of riboflavin, 50 mg of nicotinic acid, 50 mg of Ca-D-pantothenate, 1 mg of vitamin B_12_, 50 mg of 4-aminobenzoid acid, and 50 mg of lipoic acid.

Trace elements solution I, which was ×1,000 concentrated, contained per liter 50 mm HCl, 61.8 mg of H_3_BO_4_, 99.0 mg of MnCl_2_·4H_2_O, 1.49 g of FeCl_2_·4H_2_O, 119 mg of CoCl_2_·6H_2_O, 23.8 mg of NiCl_2_·6H_2_O, 68.2 mg of ZnCl_2_, and 17.0 mg of CuCl_2_·2H_2_O. Trace elements solution II, which was ×1,000 concentrated, contained per liter 10 mm NaOH, 17.3 mg of Na_2_SeO_3_, 33.0 mg of Na_2_WO_4_·2H_2_O, and 24.2 mg of Na_2_MoO_4_·2H_2_O.

### Construction of E. coli ΔtalAB double knockout strain

*E. coli* BW25113, a K-12 derivative, which has been used for the Keio collection, was used to make the double transaldolase knockout, as was done by Nakahigashi *et al.* (18). First, the strain was transformed with pKD46, a temperature-sensitive plasmid containing the Lambda Red recombination system and an ampicillin-resistance marker. Cells were then grown at 30 °C and transformed with a linear knockout cassette derived from pKD3, containing a kanamycin-resistance marker flanked by *FRT* sites and 50-base pair arms homologous to the chromosome, such that *talB* would be removed, save for the start codon, and the last seven codons. The knockout cassette was generated from pKD4 with primers AGACCGGTTACATCCCCCTAACAAGCTGTTTAAAGAGAAATACTATCATGGTGTAGGCTGGAGCTGCTTC and GACCGACTTCCCGGTCACGCTAAGAATGATTACAGCAGATCGCCGATCATCATATGAATATCCTCCTTAGTTCCTATTCC. Transformed cells were grown on LB + kanamycin, at 37 °C, to select mutants and simultaneously cure pKD46. Following the selection of a correct mutant, pCP20, a temperature-sensitive plasmid containing the yeast flippase (flp) recombinase gene was transformed to remove the kanamycin marker from the genome by recombining the *FRT* sites. pCP20 was cured by growing the cells at 37 °C. The whole process was repeated to remove the *talA* gene as well. Primers GAATTAACGCACTCATCTAACACTTTACTTTTCAAGGAGTATTTCCTATGGTGTAGGCTGGAGCTGCTTC and TTCGGGACATATAACACTCCGTGGCTGGTTTATAGTTTGGCGGCAAGAAGCATATGAATATCCTCCTTAGTTCCTATTCC were used to generate the linear knockout cassette for *talA*, using pKD4 as a template.

### Plasmid construction and heterologous expression of 6-phosphofructokinases

The two 6-phosphofructokinases of *C. thermosuccinogenes* (CDQ83_11320 and CDQ83_07225) were cloned previously into pET-28b(+) (24). The 6-phosphofructokinase of *E. coli* (BW25113_3916) was cloned in identical fashion using primers TACTTCCAATCCAATGCAATTAAGAAAATCGGTGTGTTGACAAGC and TTATCCACTTCCAATGTTAATACAGTTTTTTCGCGCAGTCC. The pET-28b(+)-based vectors were constructed in *E. coli* DH5α and then transformed to *E. coli* Rosetta for heterologous expression.

The pyrophosphate-dependent 6-phosphofructokinase of *C. thermocellum* strain DSM 1313 (locus_tag *Clo1313_1876*) was amplified using primers XSH0718 (sequence 5′-CATCACCACCACCACCATATGAGCCGTTTAGAAGGTG-3′) and XSH0719 (sequence 5′-GCGGCCGCGAGACCCTAACCTTATTTTCTTGCAAGAACC-3′), and then cloned into the pD861-NT expression vector (DNA 2.0 Inc., Menlo Park, CA) using isothermal assembly (36), to create plasmid pSH157. The assembled plasmid pSH157 was then cloned into T7 Express *lysY*/*Iq* Competent *E. coli* (New England Biolabs catalog number C3013I), using 50 μg/ml of kanamycin for selection of transformants.

Expression of the 6-phosphofructokinases was done by growing the *E. coli* strains in 0.5–2 liters of LB medium containing the appropriate antibiotics up to an OD_600_ of around 0.6, after which the cultures were place on ice for 20 min and expression was induced with 0.2 mm isopropyl 1-thio-β-d-galactopyranoside for the pET-28b(+)-based vectors and with 4 mm rhamnose for the pD861-NT-based vector. Cells were grown for another 3–4 h at 37 °C, after which the cells were harvested for protein purification.

### Metabolite extraction for MS

Biomass was isolated by rapid vacuum filtration of 5–20 ml of culture broth, adapted from the method by Sander *et al.* (37). For this, 0.2-μm nylon filters (Whatman®) were used. After filtration, the filter was immediately placed upside down in 3 to 10 ml of solvent, which was kept in a polystyrene Petri dish (50 mm diameter, Falcon®) placed on an aluminum block precooled at −80 °C. The extraction solvent consisted of a mixture of acetonitrile, methanol, and water, mixed at a ratio of 2:2:1 (v/v). The filter was kept in the extraction solvent for 5 min, after which the extract was kept on dry ice until transferred to a freezer for storage at −80 °C. All aliquots were stored until being further processed in low protein binding collection tubes (Thermo Scientific^TM^). The entire process was carried out aerobically.

20 ml of the *E. coli* cultures grown in M9 medium at OD_600_ of 0.5–0.8 was used, in combination of 5 ml of extraction solvent. For *C. thermosuccinogenes*, up to 10 ml of culture was used, depending on the OD_600_, in combination with 3 ml of extraction solvent. The higher the OD_600_, the smaller the culture that could be filtered without clogging the filter. It is not clear why cellulolytic clostridia cultures tend to clog the filters so quickly.

### HILIC mass spectrometry for detection of sedoheptulose 7-phosphate and sedoheptulose 1,7-bisphosphate in metabolite cell extracts

Identification of sedoheptulose 7-phosphate and sedoheptulose 1,7-bisphosphate was performed using a LC-MS setup as described by Schatschneider *et al.* (38). Briefly, LC-Orbitrap-MS analysis was performed using an ACQUITY UPLC M-Class chromatography system coupled to a high-resolution Orbitrap mass spectrometer (Q-Exactive plus, Thermo Fisher Scientific). For chromatographic separation, a ZIC-HILIC column (1.0 × 150 mm, 5-μm particle size, Merck KGaA, Germany) was operated at room temperature using 20 mm ammonium carbonate in water (pH 9.1) as mobile phase A and 100% acetonitrile as mobile phase B. A gradient was maintained at 40 μl/min from 25% A to 55% A over 15 min and further to 30% A over 2.5 min. Samples were taken from −80 °C immediately before injection, brought to 4–8 °C on ice, and mixed with injection buffer (85% solvent B in solvent A, including 20 mm sodium citrate) at a ratio of 1:1 (v/v). The reaction mixture was centrifuged at 14,000 rpm for 3 min at 4–8 °C and 2.5 μl were subsequently injected onto the separation column. The mass spectrometer was operated alternating in full scan and PRM mode. Full scan was acquired from 260–700 *m*/*z* in ESI negative mode (−2.5 kV), at a resolution of 70,000. Parallel reaction monitoring was performed for *m*/*z* 289.03 ([M-H]^−^, S7P) and 368.99 ([M-H]^−^, S1,7BP) precursor ions at an isolation window of 2.0 *m*/*z*. High-energy collisional dissociation fragmentation was performed using a NCE of 27 and which fragment ions were measured at a resolution of 17,000. Raw data were analyzed using XCalibur 4.1 (Thermo) and areas were integrated using Skyline 4.1.0. The fragmentation pattern and elution time for sedoheptulose 7-phosphate was compared with a commercial standard (Sigma Aldrich) and the peak obtained from the *E. coli* double transaldolase mutant and WT metabolite extracts were used as control. The fragmentation pattern and elution position for sedoheptulose 1,7-bisphosphate was compared with the metabolite extract *E. coli* Δ*talAB* (see above).

### In vitro phosphofructokinase assays with analysis by MS

#### 

##### Protein purification

50 ml of LB cultures of *E. coli* strains overexpressing the *C. thermocellum* or *C. thermosuccinogenes* PP_i_-PFK were grown, induced, and harvested as described above.

Protein purification for the purposes of demonstrating *in vitro* conversion of sedoheptulose 7-phosphate to sedoheptuoloase 1,7-bisphosphate by the purified PP_i_-PFK in the presence of PP_i_ was performed as previously described (39). To obtain purified PP_i_-PFK protein, the induced *E. coli* cells were resuspended in 100 mm Tris-HCl buffer (pH 7 at 55 °C). Approximately 70,000 units of Readylyse enzyme (Lucigen catalog number R1802) was added to the cell suspension, and the mixture was incubated for 10 min at room temperature. 5 units of DNase I (Thermo Fisher Scientific catalog number 90083) were then added to reduce the viscosity of the cell lysate; the sample was incubated for another 10 min at room temperature. The resulting solution was at >20,000 × *g* for 5 min, and the cell extract supernatant was used in future steps. *E. coli* proteins in the cell extract were denatured by incubating the cell extract at 55 °C for 30 min; the denatured proteins were removed by centrifugation at >20,000 × *g* for 5 min. His tag purification of the PP_i_-PFKs from the heat-treated cell extracts was done using the HisSpinTrap kit (GE Healthcare catalog number 28-4013-53). Eluted PP_i_-PFK proteins were desalted using a 10-kDa molecular mass cutoff filter (Millipore catalog number UFC501024) and 100 mm Tris-HCl buffer, to reduce the imidazole concentration to <1 mm.

##### Assay conditions

Enzyme assays were performed in an anaerobic chamber, with an atmospheric composition of 85% nitrogen, 10% carbon dioxide, and 5% hydrogen. Assay chemicals were purchased from Sigma Aldrich. All samples were incubated at 55 °C in a heat block for the entirety of the experiment. Assay reaction composition was based off previously described assay conditions, and comprised 100 mm Tris-HCl (pH 7 at 55 °C), 5 mm MgCl_2_, 5 mm sodium PP_i_, 1 mm of either sedoheptulose 7-phosphate or fructose 6-phosphate, 4 units of rabbit aldolase where used (Sigma catalog number A8811), and purified PP_i_-PFK protein (see next paragraph for more information on enzyme loading). In all cases, the reactions were started upon addition of sodium PP_i_, or an equivalent volume of buffer for assay reactions that did not contain PP_i_. The initial assay volume was 400 μl.

The specific PP_i_-PFK activity was first determined for each of the purified PP_i_-PFKs on the day of the experiment. The amount of purified PP_i_-PFK used for the assay was then adjusted for each sample and replicate, such that each assay reaction would contain an enzyme loading corresponding to ∼0.01 μmol/min of PP_i_-PFK activity.

Samples from the enzyme reactions were collected in the following manner: the tube containing a given assay mixture was removed from the heat block and briefly vortexed to mix the contents. 20 μl of assay mixture was then collected and then quickly added to 80 μl of very cold (≤ −30 °C) 1:1, acetonitrile:methanol mixture to quench enzyme activity, and then vortexed briefly to mix; quenching solution was kept at ≤ −30 °C by putting them in contact with a metal heat block sitting atop a 4-inch thick aluminum block, both of which had been pre-chilled at −80 °C for at least 48 h prior to use (40). The quenched sample was then stored at −80 °C until analysis. Standards of S7P, F6P, FBP, E4P, and DHAP at three different concentrations each were also prepared to allow for quantification of these compounds in the assay samples.

##### Mass spectrometry analyses of assay samples

Assay samples were analyzed as previously described (39), using an LC-MS/MS system a Thermo Scientific Vanquish UHPLC coupled by heated electrospray ionization (HESI) to a hybrid quadrupole-high resolution mass spectrometer (Q Exactive Orbitrap, Thermo Scientific). Liquid chromatography separation was performed using an ACQUITY UPLC BEH C18 (2.1 × 100 mm column, 1.7-μm particle size), with a flow rate of 0.2 ml/min. For the instrument run, Solvent A was 97:3, water:methanol with 10 mm tributylamine and ∼9.8 mm acetic acid (pH ∼ 8.2); solvent B was 100% methanol. Total run time was 24.5 min with the following gradient: 0 min, 5% B; 2.5 min, ramp from 5% B to 95% B over 14.5 min; hold at 95% B for 2.5 min; return to 5% B over 0.5 min; hold at 5% B for 5 min. MS scans consisted of full negative mode MS scanning for *m*/*z* between 70 and 1000 from time 0 to 18.5 min. Sample preparation involved first evaporating the solvents with a nitrogen blowdown evaporator, and then resuspending the dried samples in Solvent A.

Metabolite peaks were identified using the open source software, El-MAVEN (46) (https://elucidatainc.github.io/ElMaven/)^5^. Response factors for S7P, F6P, FBP, E4P, and DHAP standards were used to determine the concentrations of these five compounds in the assay samples.

### Phosphofructokinase kinetics assays

The harvested cells were washed with cold 50 mm MOPS buffer (pH 7.4 at room temperature) containing 20 mm imidazole and resuspended in the same buffer with cOmplete^TM^, mini, EDTA-free protease inhibitor mixture (Roche) added; 1 tablet per ∼10 ml. Cells were lysed using a French press at ∼120 kilopascal. Lysate was centrifuged at 20,000 × *g* for 10 min at 4 °C. A HisTrap^TM^ HP column (GE Healthcare, optimal at pH 7.4) with an ÄKTA pure FPLC system were used for the purification. Elution was done over a gradient with 50 mm MOPS buffer (pH 7.4 at room temperature) containing 500 mm imidazole. The buffer of the eluted protein was then exchanged with 50 mm MOPS (pH 7.0 at room temperature) using an Amicon® ultracentrifugal filter (Merck) with a nominal molecular mass limit of 10,000 Da. SDS-PAGE was used to verify purity.

The 6-phosphofructokinase assay was adapted from Zhou *et al*. (25) and contained 50 mm MOPS (pH 7.0 at room temperature), 5 mm MgCl_2_, 2 mm ATP or 1 mm pyrophosphate, 0.15 mm NADH, 4 units/ml of aldolase (lyophilized, rabbit), and 2 units/ml of glycerol-3-phosphate dehydrogenase (lyophilized, rabbit). The reaction was carried out at 55 °C. Fructose 6-phosphate or sedoheptulose 7-phosphate (Ba-salt, Carbosynth) was added to start the reaction, at varied concentrations. The final volume was 1 ml for reactions with fructose 6-phosphate and 0.5 ml for reactions with sedoheptulose 7-phosphate. For the PfkA of *E. coli*, 0.25 mm ADP was added to the assay, as it is known to be an allosteric activator (41). For the ATP/GTP-dependent 6-phosphofructokinases of *C. thermosuccinogenes*, 20 mm NH_4_Cl_2_ was added to the reaction, as it was found to be an absolute requirement for its activity. Previously, auxiliary enzymes from an ammonium sulfate suspension were used (24), but with sedoheptulose 7-phosphate as a Ba-salt, this was not possible due to precipitation of BaSO_4_, which is how we found out that the enzyme requires ammonium. The reaction was followed in a Hitachi U-2010 spectrophotometer with a thermoelectric cell holder, by measuring the decreasing absorbance of reduced NADH at 340 nm. The reaction was run up to 5 min and a window of 10 to 40 s was used for determining the initial rates.

The Michaelis-Menten equation and the Hill equation were fitted to the data by minimizing the sum of the squares of the vertical differences, to find *K_m_*/*K*_½_, *k*_cat_, and *n*. The data and the fitted models can be found in the Figs. S2–S5.

## Author contributions

J. G. K., S. H., L. R. L., D. G. O., and R. v. K. conceptualization; J. G. K., S. H., M. P., R. H., D. M. S., J. C., and D. A.-N. investigation; J. G. K., S. H., and M. P. visualization; J. G. K., S. H., M. P., R. H., D. M. S., J. C., D. A.-N., L. R. L., D. G. O., and R. v. K. methodology; J. G. K. and S. H. writing-original draft; J. G. K., S. H., M. P., D. A.-N., L. R. L., D. G. O., and R. v. K. writing-review and editing; L. R. L. and R. v. K. resources; L. R. L., D. G. O., and R. v. K. supervision.

## Supplementary Material

Supporting Information

## Footnotes

4 “Cellulolytic clostridia” refers to a large group of mesophilic and thermophilic bacteria of which most are able to grow on (hemi)cellulosic substrates, and of which *C. thermocellum* and *C. cellulolyticum* are probably the most well-studied. Recently, they have been placed within a newly named family called *Hungateiclostridiaceae*, containing Genus names such as *Hungateiclostridium* and *Pseudoclostridium* (42), and for a brief time when some were also referred to cellulolytic clostridia as the *Ruminiclostridium* genus (43, 44). However, even more recently, the family name *Hungateiclostridiaceae* and the corresponding Genus names were suggested to be illegitimate and placement within the *Acetivibrio* genus was proposed (45). In light of this ongoing discussion, we have decided to refer to them as cellulolytic clostridia, and use the commonly used names *C. thermocellum* and *C. thermosuccinogenes* for the organisms of interest in this study (instead of *Hungateiclostridium thermocellum* and *Pseudoclostridium thermosuccinogenes*). Note that *C. thermosuccinogenes* ferments a wide range of C_5_ and C_6_ sugars, and sugar polymers, but not crystalline cellulose.

5 Please note that the JBC is not responsible for the long-term archiving and maintenance of this site or any other third party hosted site.

## Footnotes

3 The abbreviations used are:

PPP: pentose phosphate pathway
SBP: sedoheptulose 1,7-bisphosphate
DHAP: dihydroxyacetone phosphate
PKP: phosphoketolase pathway
PFK: phosphofructokinase
F6P: fructose 6-phosphate
S7P: sedoheptulose 7-phosphate
E4P: erythrose 4-phosphate
G3P: glyceraldehyde 3-phosphate.
