# Supplementary material for: The pentose phosphate pathway of cellulolytic clostridia relies on 6-phosphofructokinase instead of transaldolase
Source: J Biol Chem. 2019 Dec 22;295(7):1867–78. doi: 10.1074/jbc.RA119.011239 (PMC7029132; doi:10.1074/jbc.RA119.011239)
Supplement: Supporting Information [file supp_295_7_1867__index.html]

The pentose phosphate pathway of cellulolytic clostridia relies on 6-phosphofructokinase instead of transaldolase — PPP of cellulolytic clostridia relies on PFK — The pentose phosphate pathway of cellulolytic clostridia relies on 6-phosphofructokinase instead of transaldolase — PPP of cellulolytic clostridia relies on PFK — Supporting Information 

# The pentose phosphate pathway of cellulolytic clostridia relies on 6-phosphofructokinase instead of transaldolase

## Supporting Information

- Supporting Information PDF - Fitted models of PFK kinetics assays
