# Supplementary material for: The pentose phosphate pathway of cellulolytic clostridia relies on 6-phosphofructokinase instead of transaldolase
Source: J Biol Chem. 2019 Dec 22;295(7):1867–78. doi: 10.1074/jbc.RA119.011239 (PMC7029132; doi:10.1074/jbc.RA119.011239)
Supplement: Supporting Information [file supp_RA119.011239_156101_2_supp_447520_q2tbz1.pdf]

Supporting information

The pentose phosphate pathway of cellulolytic clostridia relies on 6-phosphofructokinase instead of transaldolase

Jeroen G. Koendjbiharie, Shuen Hon, Martin Pabst, Robert Hooftman, David M. Stevenson, Jingxuan Cui, Daniel Amador-Noguez, Lee R. Lynd, Daniel G. Olson, Richard van Kranenburg

Contents

Negative control *In vitro* phosphofructokinase assays S2
Figure S1 ..... S2
Fitted kinetic models S3
Figure S2 ..... S3
Figure S3 ..... S4
Figure S4 ..... S5
Figure S5 ..... S6

Negative control *In vitro* phosphofructokinase assays

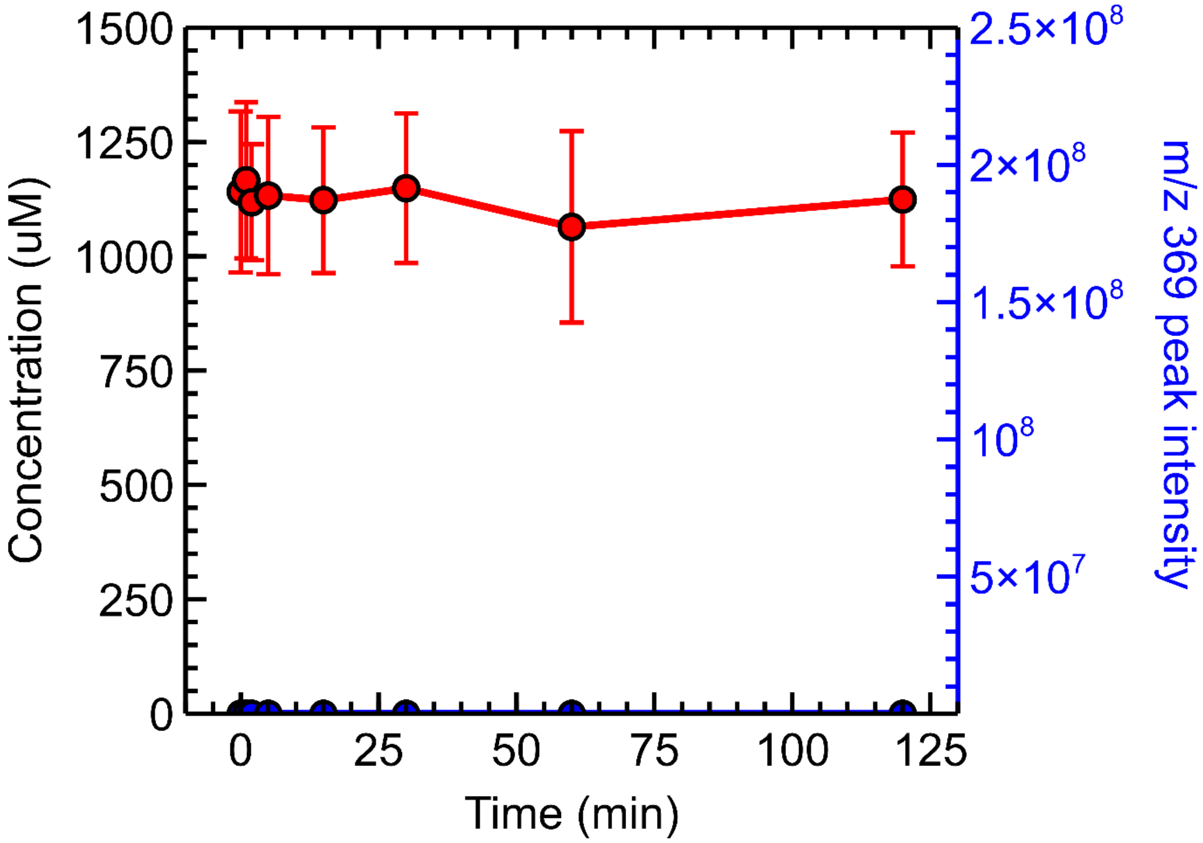

Figure S1: Negative control of the *In vitro* time-course assays using sedoheptulose-7-phosphate (red), lacking added enzyme. In blue the peak intensity at a m/z of 369, corresponding to sedoheptulose-1,7-bisphosphate. Error bars represent one standard deviation ($n \geq 2$).

Fitted kinetic models

Clostridium thermosuccinogenes PPi-PFK

Michaelis-Menten kinetics

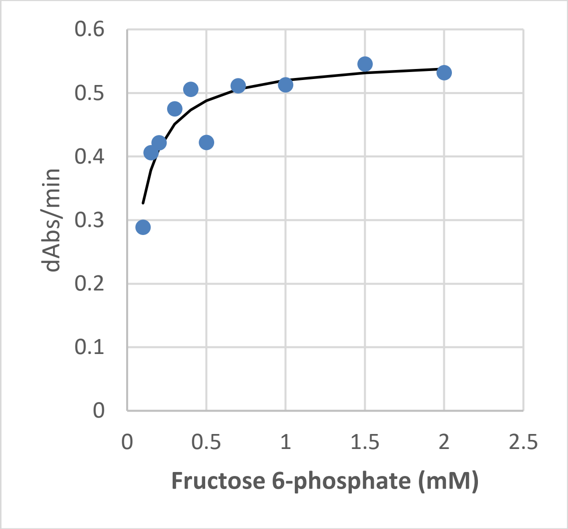

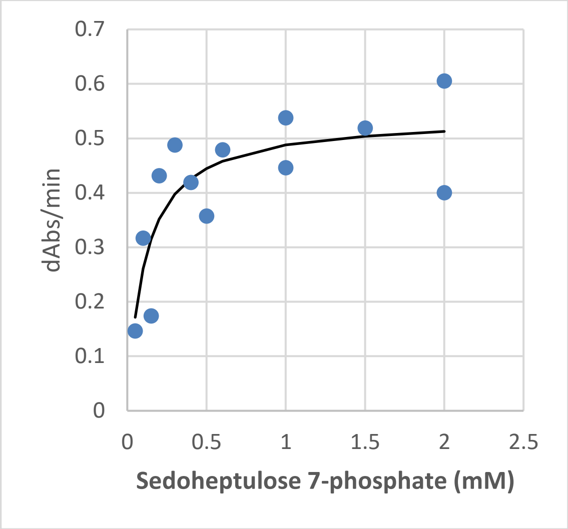

F6P: K_M = 0.07045054 mM, V_{max} = 0.556745262 dAbs/min

S7P: K_M = 0.107440985 mM, V_{max} = 0.540231693 dAbs/min

Hill kinetics

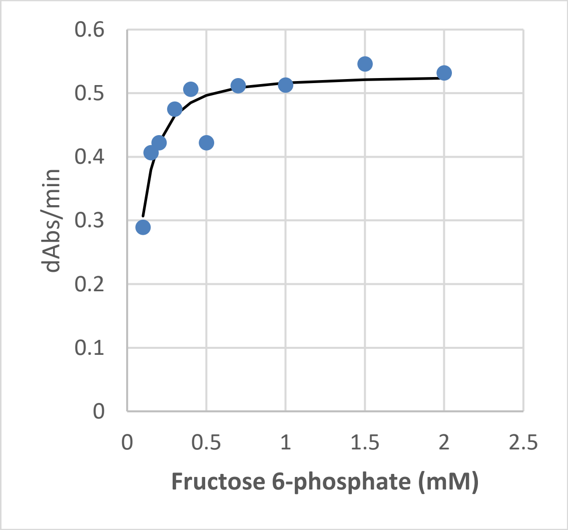

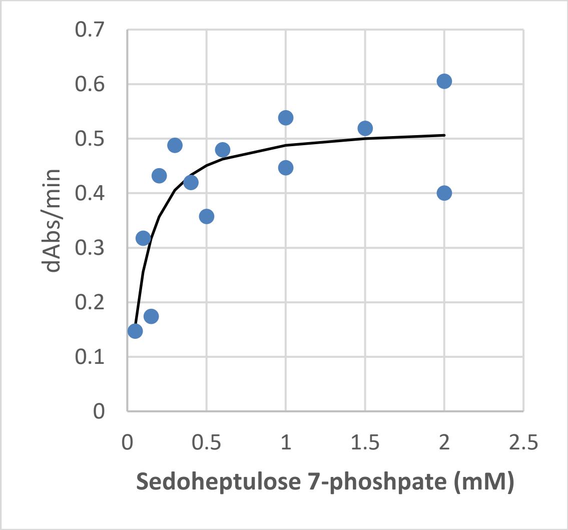

F6P: $K_{1/2}$ = 0.080468265 mM, V_{max} = 0.527619413 dAbs/min, n = 1.51508789

S7P: $K_{1/2}$ = 0.103684272 mM, V_{max} = 0.521954034 dAbs/min, n = 1.170962878

Figure S2: Data of the kinetics assays using *Clostridium thermosuccinogenes* PPi-6-phosphofructokinase (PFK) for the conversion of fructose 6-phosphate (F6P) and sedoheptulose 7-phosphate (S7P). Including fitted models of both Michealis-Menten kinetics and Hill kinetics.

Clostridium thermocellum PPi-PFK

Michaelis-Menten kinetics

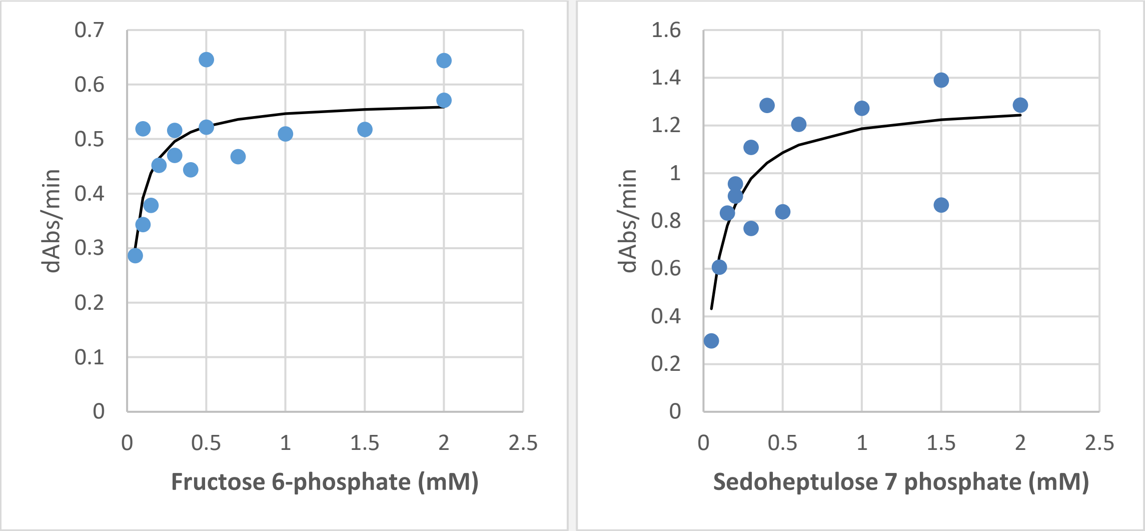

F6P: K_M = 0.046004018 mM, V_{max} = 0.571547641 dAbs/min

S7P: K_M = 0.10131141 mM, V_{max} = 1.306125685 dAbs/min

Hill kinetics

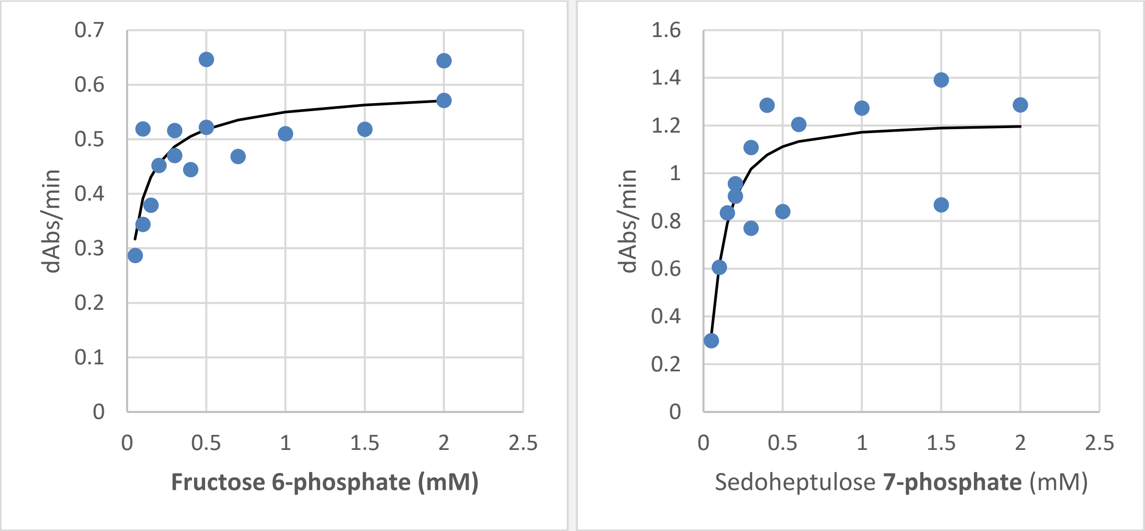

F6P: $K_{1/2}$ = 0.04393566 mM, V_{max} = 0.605090926 dAbs/min, n = 0.735169235

S7P: $K_{1/2}$ = 0.098186063 mM, V_{max} = 1.20981373 dAbs/min, n = 1.491188053

Figure S3: Data of the kinetics assays using *Clostridium thermocellum* PPi-6-phosphofructokinase (PFK) for the conversion of fructose 6-phosphate (F6P) and sedoheptulose 7-phosphate (S7P). Including fitted models of both Michealis-Menten kinetics and Hill kinetics.

E. coli PfkA

Michaelis-Menten kinetics

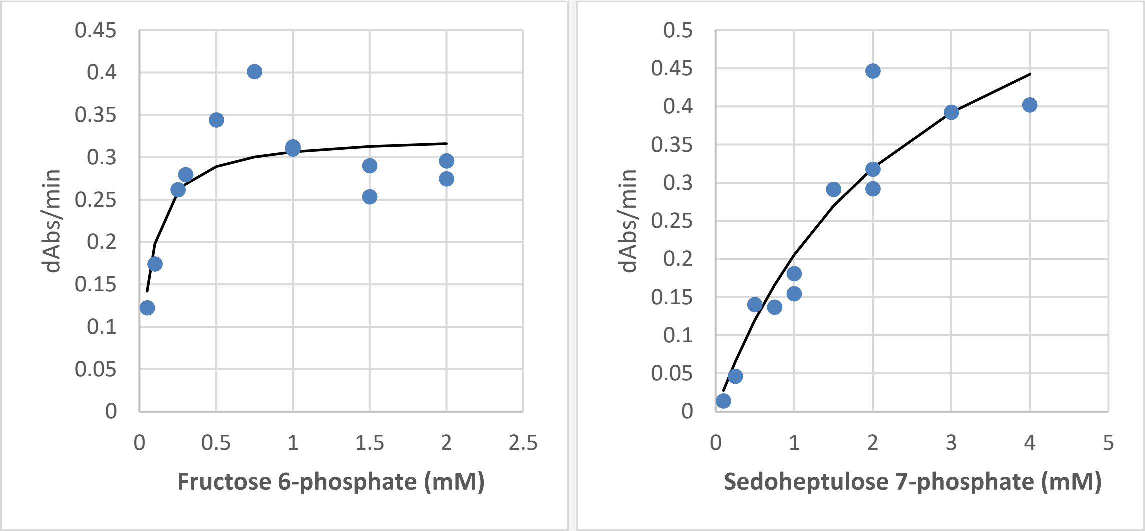

F6P: K_M = 0.064895717 mM, V_{max} = 0.326490009 dAbs/min

S7P: K_M = 2.489181822 mM, V_{max} = 0.717467989 dAbs/min

Hill kinetics

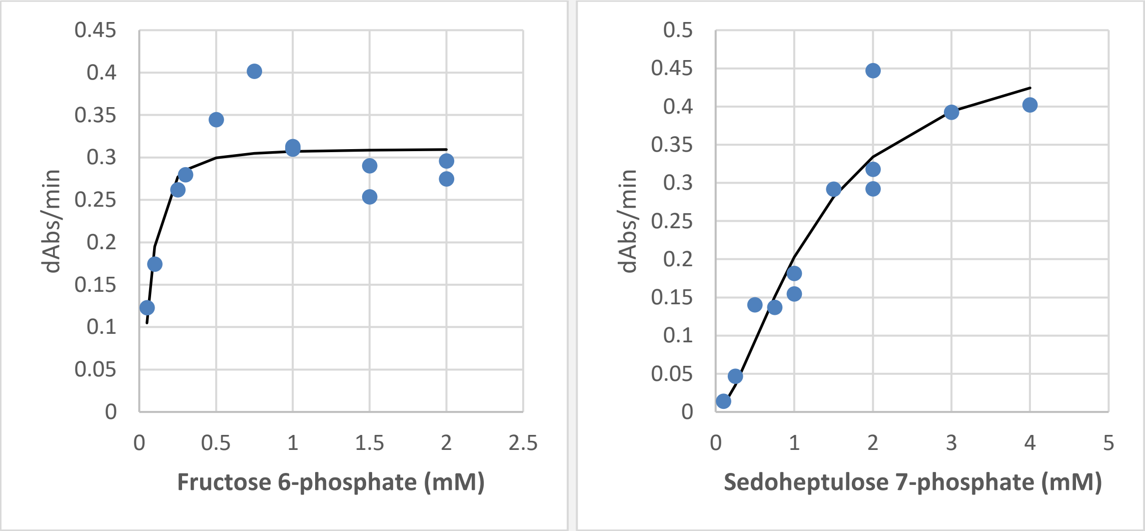

F6P: $K_{1/2}$ = 0.073763326 mM, V_{max} = 0.310312305 dAbs/min, n = 1.729659661

S7P: $K_{1/2}$ = 1.24179394 mM, V_{max} = 0.48943044 dAbs/min, n = 1.603138135

Figure S4: Data of the kinetics assays using *E. coli* 6-phosphofructokinase (PfkA) for the conversion of fructose 6-phosphate (F6P) and sedoheptulose 7-phosphate (S7P). Including fitted models of both Michealis-Menten kinetics and Hill kinetics.

P. thermosuccinogenes ATP/GTP-PFK

Michaelis-Menten kinetics

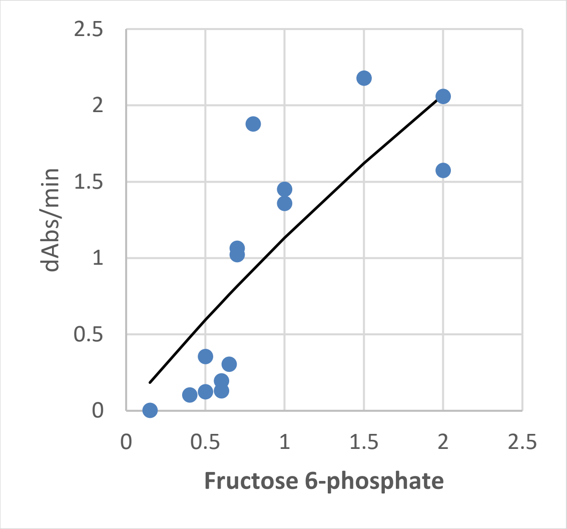

F6P: K_M = 9.550905907 mM, V_{max} = 11.94361907 dAbs/min

S7P: No activity

Hill kinetics

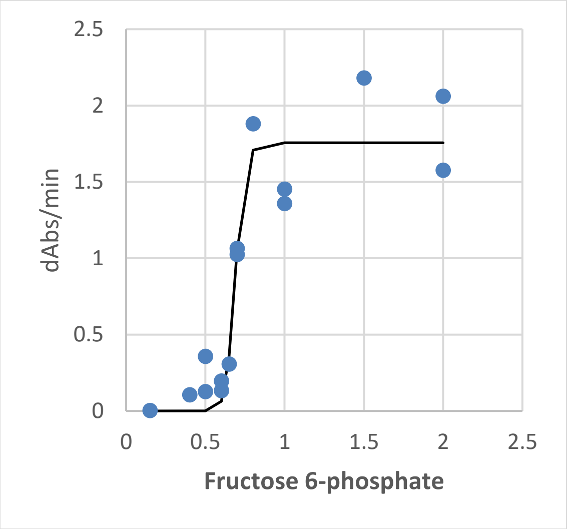

F6P: $K_{1/2}$ = 0.68836161 mM, V_{max} = 1.756115449 dAbs/min, n = 23.87224309

S7P: No activity

Figure S5: Data of the kinetics assays using *Clostridium thermosuccinogenes* ATP/GTP-6-phosphofructokinase (PFK) for the conversion of fructose 6-phosphate (F6P) and sedoheptulose 7-phosphate (S7P). Including fitted models of both Michealis-Menten kinetics and Hill kinetics.
